# Extending the breadth of saliva metabolome fingerprinting by smart template strategies and effective pattern realignment on comprehensive two-dimensional gas chromatographic data

**DOI:** 10.1007/s00216-023-04516-x

**Published:** 2023-01-12

**Authors:** Simone Squara, Friederike Manig, Thomas Henle, Michael Hellwig, Andrea Caratti, Carlo Bicchi, Stephen E. Reichenbach, Qingping Tao, Massimo Collino, Chiara Cordero

**Affiliations:** 1 Dipartimento Di Scienza E Tecnologia del Farmaco, Università Degli Studi Di Torino, Via Pietro Giuria 9, 10125 Turin, Italy; 2 Food Chemistry, Technische Universität Dresden, Dresden, Germany; 3 Special Food Chemistry, Technische Universität Dresden, Dresden, Germany; 4 Computer Science and Engineering Department, University of Nebraska, Lincoln, NE USA; 5 GC Image LLC, Lincoln, NE USA; 6 Dipartimento Di Neuroscienze “Rita Levi Montalcini”, University of Turin, Turin, Italy

**Keywords:** Comprehensive two-dimensional gas chromatography, Smart template functions, Saliva metabolomics, 2D pattern realignment, 2D data normalization, UT fingerprinting

## Abstract

Comprehensive two-dimensional gas chromatography with time-of-flight mass spectrometry (GC × GC-TOFMS) is one the most powerful analytical platforms for chemical investigations of complex biological samples. It produces large datasets that are rich in information, but highly complex, and its consistency may be affected by random systemic fluctuations and/or changes in the experimental parameters. This study details the optimization of a data processing strategy that compensates for severe 2D pattern misalignments and detector response fluctuations for saliva samples analyzed across 2 years. The strategy was trained on two batches: one with samples from healthy subjects who had undergone dietary intervention with high/low-Maillard reaction products (dataset A), and the second from healthy/unhealthy obese individuals (dataset B). The combined untargeted and targeted pattern recognition algorithm (i.e., UT fingerprinting) was tuned for key process parameters, the signal-to-noise ratio (S/N), and MS spectrum similarity thresholds, and then tested for the best transform function (global or local, affine or low-degree polynomial) for pattern realignment in the temporal domain. Reliable peak detection achieved its best performance, computed as % of false negative/positive matches, with a S/N threshold of 50 and spectral similarity direct match factor (DMF) of 700. Cross-alignment of bi-dimensional (2D) peaks in the temporal domain was fully effective with a supervised operation including multiple centroids (reference peaks) and a *match-and-transform* strategy using affine functions. Regarding the performance-derived response fluctuations, the most promising strategy for cross-comparative analysis and data fusion included the mass spectral total useful signal (MSTUS) approach followed by *Z-*score normalization on the resulting matrix.

**Supplementary Information:**

The online version contains supplementary material available at 10.1007/s00216-023-04516-x.

## Introduction

The use of saliva in clinical diagnostics is historically under-explored compared to other bio-fluids such as urine or blood. However, emerging biotechnologies and salivary diagnostics have extended the range of saliva-based diagnostics from the oral cavity, such as for periodontal diseases and caries risk, to the physiological status, due to its biological equilibrium with plasma [1]. It has been shown that saliva metabolites closely resemble metabolic changes that take place in blood and may therefore reflect a variety of pathophysiological and nutritional changes, as well as exposure to medications and environmental factors [2–5].

The *-omic* analytical strategies aim to capture the variety of encrypted information of complex samples to enable to the higher level information related to the phenomena under study. Well-established methodologies in metabolomics, i.e., *profiling* and *fingerprinting*, have been developed as separate analytical techniques/approaches capable of informing about compositional differences between samples [6, 7]. If analytes of interest are identified upfront and tracked across all samples, profiling can be done specifically. However, the procedure can be extended toward a thorough evaluation of all detected components and referred to as *untargeted* profiling [8]. Fingerprinting, on the other hand, is a high-throughput approach that may identify compositional changes between samples [9–11]; however, it may not always produce precise quantitative data or provide analyte identification for all of the constituents. The goal of fingerprinting is to extract the non-obvious chemical information present in the entire instrumental signal. Chemometrics and multivariate analysis allow then the access to higher level information and understanding. In the case of comprehensive two-dimensional chromatography, fingerprinting acquires a new meaning: it is referred to as *chromatographic fingerprinting* and corresponds to a pattern recognition procedure where detected features are annotated and tracked across many samples based on their relative retention and spectral similarity [6].

Comprehensive two-dimensional gas chromatography coupled with time-of-flight mass spectrometry (GC × GC-TOFMS) is one of the most informative analytical platforms for complex sample analysis. It allows detailed profiling and high-resolution chromatographic fingerprinting of biofluids thanks to the high separation power, chromatographic resolution, and sensitivity. The benefits are achieved by combining two separation dimensions interfaced by a thermal modulator, which allows for an efficient compression in space of the injection band before the ^2^D separation [12–14]. However, to implement the fingerprinting approach in long-term studies, pattern realignment strategies and response normalization are needed; otherwise, data acquired with different analytical setups and/or with variable detection performances cannot be cross-compared [6, 15, 16].

This is crucial because biobanks—a term we use broadly to cover a range of structured collections, including biorepositories and databases—have become central engines of biomedical research and there are a growing number of biobanks built by directly collecting data and samples from a population or subpopulation to assemble a large-scale research resource. The re-evaluation and reanalysis of archived samples have become inevitable, as technology, bioinformatics pipelines, and medical knowledge evolve and expand over time. Both the issues, normalization of samples from different subpopulations or collection kinetics and the need for sample reanalysis, are pressing concerns, which deserve better elucidation.

In the challenging context of 2D pattern realignment between datasets, the *smart template* concept [13, 17] based on pattern recognition algorithms was demonstrated to be highly reliable and effective. The smart template method was developed to align 2D peaks and *peak regions* across many samples using a reference pattern (i.e., the template). It employs rule-based constraints (e.g., retention times windows and multispectral matching) to increase matching accuracy. Each reference peak in a smart template has constraint rules, including the spectral similarity value (NIST algorithm [18]) vs. the reference peak spectral signature, as well as arithmetic and logical operators applied to the MS spectrum. Constraints could be related to the relative intensity of a single or multiple *m*/*z* fragments, and to the identification of the base peak or molecular peak [19]. If constraint rules are verified, peaks and peak regions from the reference pattern (template) are realigned to putative candidates in an analyzed pattern. Correspondences are established, thanks to the pattern transform, even in the presence of retention times shifts or temporal inconsistencies [6, 16, 20].

In particular, if misalignments are induced by changes in oven temperature programming, they can be successfully compensated by an affine transformation, an algorithm for template matching suitable for intra-batch cross-comparative analysis [17]. On the other hand, larger misalignments such as those generating non-linear changes in retention times across the analyses (i.e*.*, variations in the actual pressure drop [16, 21, 22], adoption of different modulation principles [23, 24], changes in the modulation period *P*_M_ [16, 25]) can be successfully treated by global, low-degree polynomial transforms [17, 22, 26].

However, in a scenario of random pattern misalignments, generated by the combination of multiple concurrent variables (i.e*.*, pressure drop, column set-up, *P*_M_), supervision and manual operations may be necessary to guide the algorithm-based realignment. The primary goal of this study was to define and validate a simple, yet effective, strategy to guide template matching algorithms for a combined untargeted and targeted (*UT*) fingerprinting [8, 27–29] exploration on datasets with severe misalignments. In addition, as a secondary objective, different response normalization methods were tested to allow for effective dataset comparative analysis. As a challenging bench test, the saliva metabolome was selected. The two salivary datasets considered were acquired in a 2-year time frame with different analytical setups. Dataset “A,” acquired in February 2020, relates to a proof-of-concept diet intervention study with food rich in Maillard reaction products (MRPs) [30], while dataset “B,” acquired in November 2017, relates to a study on two different populations of subjects with severe obesity with normal or altered metabolic parameters [14].

## Materials and methods

### Reference compounds and solvents

A pure standard solution of *n‐*alkanes (from *n‐*C7 to *n‐*C30) for linear retention indices (*I*^*T*^) calibration and system quality control was from Merck (Milan, Italy) and prepared in toluene at the concentration of 100 mg L^−1^.

The internal standard (IS) 1,4-dibromobenzene (from Merck, Milan, Italy) solution was prepared in toluene at a concentration of 10 g L^−1^.

Pure reference standards used for identity confirmation of pyruvic acid, lactic acid, malonic acid, succinic acid, malic acid, 2-ketoglutaric acid, 3-hydroxybutyric acid, fumaric acid, 2-keto-3-metilvaleric acid, aspartic acid, hippuric acid, citric acid, uric acid, l-alanine, l-valine, l-leucine, l-proline, glycine, l-threonine, l-tyrosine, l-phenylalanine, l-isoleucine, l-methionine, l-cysteine, l-ornithine, l-tryptophan, xylitol, ribitol, fructose, galactose, glucose, mannitol, myo-inositol, glycerol, palmitic acid, stearic acid, and creatinine were from Merck (Milan, Italy).

Derivatizing agents *O*-methyl hydroxylamine hydrochloride (MOX), N,O-bis(trimethylsilyl)trifluoroacetamide (BSTFA), and LC-grade pyridine, *n-*hexane, dichloromethane, and toluene used as solvents were all from Merck (Milan, Italy).

### Saliva samples

Subjects for dataset A were metabolically healthy German volunteers (3 females and 2 males) aged between 20 and 30, who ate unheated food virtually free of Maillard reaction products—MRPs (e.g., mainly vegetables, fruits, oils, and unroasted nuts) for 4 days. Sampling was performed from day 1 until day 5 in the morning and at 2 pm as well as 9 pm. The same group of subjects collected samples for another 5 days while eating their habitual diets and additionally including MRP-rich food. Fasting saliva was collected before breakfast and after brushing the teeth without using toothpaste and rinsing the mouth with water; quality control (QC) samples were collected from healthy Italian volunteers aged between 20 and 30 without dietary indications. Saliva was collected using Salivettes™ (Sarstedt, Germany), but the protocol was adapted to avoid stimulation: the device was placed at the center of the tongue and the collection time was 3 min; samples were stored at − 18 °C until the time of testing. The study was approved by the Ethics Committee of Technische Universität Dresden, Germany (reference: AZ 439112017). Details on the study are described in the reference paper by Manig et al. [31].

Patients from the Istituto Auxologico Italiano in Verbania, Italy, were recruited for dataset B [32, 33]. As part of the routine controls, individuals’ height, weight, and waist circumference were evaluated. For the current study, participants with a body mass index (BMI) of at least 40 kg m^−2^ were enrolled. Thirty-four obese men (BMI 40 kg m^−2^) were classified as either metabolically healthy (MHO, *n* = 10) or metabolically unhealthy (MUO, *n* = 24), depending on whether certain metabolic parameters, such as high fasting triglycerides (1.7 mmol L^−1^ or higher, 150 mg dL^−1^), decreased HDL cholesterol (1.03 mmol L^−1^, 40 mg dL^−1^), whether they were taking antihypertensive medication, had high blood pressure (130 mmHg systolic or 85 mmHg diastolic), or had fasting glucose levels above 5.6 mmol/L (100 mg dL^−1^). QC samples were collected from healthy Italian volunteers aged between 20 and 30 without dietary indications. The experimental procedure was approved by the ad hoc Ethical Research Committee of the Istituto Auxologico Italiano (Verbania, Italy). Written informed consent was obtained from the patients. The study protocol conformed to the guidelines of the European Convention on Human Rights and Biomedicine concerning biomedical research. Details on the study are described in the reference paper by Cialiè Rosso et al. [14].

### Sample preparation

A standard derivatization protocol [34] was adjusted to comply with method sensitivity and metabolite coverage. In particular, 100 μL of saliva was gently dried with nitrogen before being mixed with 25 μL of MOX (20 g L^−1^ in pyridine) and allowed to react for 2 h at 60 °C preventing the formation of multiple derivatives with enols during further silylation steps. Seventy-five microliters of BSTFA was added and the solution was left at 60 °C for 1 h under a nitrogen stream. Twenty microliters of 1,4-dibromobenzene in dichloromethane 1 g L^−1^ was added as IS, and diluted in 80 μL of toluene to a final volume of 200 μL. Samples were immediately stored in − 18 °C and analyzed within 24 h after derivatization.

### *GC* × *GC-TOFMS instrument setup and conditions*

GC × GC analyses were performed on an Agilent 7890 GC chromatograph (Agilent Technologies, Wilmington DE, USA) coupled to a Markes BenchTOF Select™ mass spectrometer featuring tandem ionization (Markes International, Llantrisant, UK). The system was equipped with a two-stage KT 2004 loop-type thermal modulator (Zoex Corporation, Houston, TX) cooled with liquid nitrogen controlled by Optimode v2.0 (SRA Intruments, Cernusco sul Naviglio, Milan, Italy).

Column settings and operative conditions for dataset A were as follows: ^1^D DB5 (95% polydimethylsiloxane, 5% phenyl; 30 m, 0.25 mm *d*_*c*_, 0.25 μm *d*_*f*_), ^2^D OV1701 (86% polydimethylsiloxane, 7% phenyl, 7% cyanopropyl; 1.3 m × 0.1 mm *d*_*c*_, 0.10 μm *d*_*f*_) from J&W (Agilent, Little Falls, DE, USA). The first 0.80 m of the ^2^D column, connected in series to the ^1^D column by a silTite μ-union (Trajan Scientific and Medical, Ringwood, Victoria, Australia), was wrapped in the modulator slit and used as loop-capillary for cryogenic modulation. The carrier gas was helium at 1.3 mL min^−1^ in constant flow mode. *P*_M_ was 3.0 s operating in multi-step mode: 0–15 min. The hot jet pulse time duration was 250 ms during the first 15 min, and 350 ms during the period 15–63 min. The cold jet flow was programmed for a linear decrease from 35% of the mass flow controller (MFC) maximum flow (i.e., 40 L min^−1^) to 5% at the end of the run. The injector temperature was kept at 280 °C operating in split mode with a split ratio: 1:20. The oven temperature ramp was 60 °C (2′) to 120 °C at 10 °C min^−1^, then to 300 °C (10′) at 4 °C min^−1^; the injection volume was 2 μL.

The TOFMS acquisition parameters were as follows: tandem ionization™ acquisition at 70 and 12 eV with an acquisition rate of 50 Hz per channel within the mass range 35–750 m/*z*; the filament voltage was set at 1.7 V. The ion source and transfer line were set at 280 °C and 290 °C, respectively.

Column settings and operative conditions for dataset B were as follows: ^1^D DB5 (95% polydimethylsiloxane, 5% phenyl; 30 m, 0.25 mm *d*_*c*_, 0.25 μm *d*_*f*_), ^2^D OV1701 (86% polydimethylsiloxane, 7% phenyl, 7% cyanopropyl; 2 m × 0.1 mm *d*_*c*_, 0.10 μm *d*_*f*_) from J&W (Agilent, Little Falls, DE, USA). The first 0.80 m of the ^2^D column, connected in series to the ^1^D column by a silTite μ-union (Trajan Scientific and Medical, Ringwood, Victoria, Australia), was wrapped in the modulator slit and used as loop-capillary for cryogenic modulation. The carrier gas was helium at 1.6 mL/min—constant flow. *P*_M_ was 5.0 s. The hot jet pulse time was 350 ms; the cold jet flow was progressively reduced with a linear function from 30% of MFC at initial conditions to 8% at the end of the run. The injector temperature was kept at 300 °C operating in split mode with a split ratio: 1:20. The oven temperature ramp was as follows: 70 °C (2′) to 120 °C at 10 °C min^−1^, then to 320 °C (1′) at 4 °C min^−1^; the injection volume was 2 μL.

TOFMS acquisition parameters were as follows: tandem ionization™ at 70 and 12 eV with an acquisition rate of 50 Hz per channel and a mass range 45–1000 m/*z*; the filament voltage was set at 1.6 V. The ion source and transfer line were both set at 290 °C respectively.

### UT fingerprinting workflow principles and parameters

A template is a pattern of 2D peaks and/or graphic objects that is built over a *reference* image(s) (single or cumulative image [35]) and then used to recognize similar patterns of 2D peaks in an *analyzed* image(s) [36]. Dedicated matching functions or transforms guide this process while effectively compensating for inconsistent retention times and variable peak detection [29]. Each template object (2D-peak/blob or peak region/area graphic) can carry various metadata including compound chemical name, retention times, mass spectra, informative ions (qualifier and quantifier ions) and their relative ratios, constraint functions to limit peak correspondences above certain thresholds, and qualifier functions.

The challenging task of multi-chromatogram fingerprinting in the presence of temporal inconsistencies and detector fluctuations was addressed successfully by introducing the concept of *peak region* features [8]. Peak regions attempt to define one chromatographic region around each individual peak thereby achieving the one-feature-to-one-analyte selectivity that is the goal of peak features approaches [8, 13], but with an implicit matching of regional features and greater robustness than can be achieved with peak detection [29]. 2D peaks and peak-regions are features adopted in the *UT* fingerprinting strategy [27, 35, 37, 38].

*UT* fingerprinting establishes a set of reliable peaks, positively matched across all or most of a set of chromatograms [39], and then uses them to align chromatograms [22] for their combination into a single, composite chromatogram. The algorithm for the determination of reliable peaks is the Consistent Cliques Method (CCM) [39] algorithm: it picks peaks that are consistently matched across several chromatograms, but possibly not all of them. The operator can define the most appropriate minimum threshold for the selection of reliable peaks: either peaks that are pairwise matched across *all but one* (most constrained) chromatogram or peaks pairwise matched in 50% + 1 (most relaxed) of the processed chromatograms. The first option will result in a fewer number but more reliable peaks, while the latter will result in a larger number but less reliable peaks. The choice of the minimum threshold is driven by the goal of the study: if realignment is the aim, the issue of a small number of reliable peaks might not be a concern as long as enough reliable peaks are found; but if the comparative analysis is the goal, certain important peaks might be lost together with the information they carry. The most constrained option does not work well for large sample sets, since even for very consistent features, the risk of a failed match increases exponentially as the number of chromatograms increases.

Once the composite chromatogram is built (i.e., the combination of the realigned responses in the 2D retention-times plane), 2D peaks are detected and their outlines are recorded to define peak-region objects. The set of reliable 2D peaks and peak-regions objects are then collected in a so-called *feature template*, or *consensus template*, covering the whole sample-set compositional diversity and capable of cross-aligning chemical feature patterns among samples [6].

Within all detected analytes (reliably matched or not over the chromatogram set), the sub-group of targeted compounds can be highlighted by completing their metadata fields (compound name, ion ratios, *I*^*T*^_*S*_) and computed together with untargeted features during the data processing.

Untargeted features comprehensively map the sample’s chemical dimensionality [40] and are automatically generated through a dedicated workflow. On the other hand, targeted features require supervised processing for the reliable identification of analytes and the definition of specific ions for response isolation and/or informative ion ratios to add constraints to the template matching process.

Identification of targeted features was carried out by matching candidate EI-MS fragmentation patterns at 70 eV (NIST MS Search algorithm, version 2.0) with those collected in commercial and in-house databases (subject to a DMF threshold of 900 and RMF threshold of 950). In addition, linear retention indices (*I*^*T*^) were adopted as a further constraint; experimental values were compared with NIST reference indices using a tolerance of ± 10 units.

### Data acquisition and processing

Data were acquired by TOF-DS software (Markes International, Llantrisant, UK) and processed using GC Image GC × GC Software ver. 2021r2 (GC Image, LLC, Lincoln, NE, USA). Data mining was performed using Matlab R2021a (The MathWorks, Inc., Natick, MA, USA) with the following packages: PCA toolbox (v1.5) [41] and Classification toolbox (v6.0) [42], and XLSTAT 2014 by Addinsoft (New York, USA).

## Results and discussion

This section illustrates the step-by-step procedure (i.e., workflow) adopted to evaluate the impact of key-processing parameters on template matching accuracy and template transformation in a context of severe misalignment. At first, template construction parameters are tested for their impact on matching accuracy (type-I and type-II errors; “Template construction parameters” section). Subsequently, the combined untargeted targeted (UT) template is applied to dataset A exploring saliva metabolite patterns correlated to a diet rich in MRPs (“Dataset A—Saliva samples from diet intervention with Maillard reaction products–rich food” section). As the third step, the UT template built on dataset A is transformed to match on dataset B retention pattern logic (“Template transformation for reliable template matching between severely misaligned patterns” section). Realigned datasets are then fused after suitable response normalization and further mined to explore the possibility of reliable re-investigation of data after their fusion (“Response normalization for consistent cross-comparison between datasets” section).

### Template construction parameters

The best combination of processing parameters to build a reliable template with consistent reference metadata (i.e., template object metadata) was first evaluated in light of its adoption as a key-tool for pattern realignment, even in a context of dramatic chromatographic shifts.

Signal-to-noise ratio (S/N) thresholds, which is the ratio of the total intensity count (TIC) of the apex spectrum to the standard deviation of background noise TIC [43], and MS similarity threshold, expressed as NIST similarity match factor (direct match factor, DMF) [18], were first considered since both contribute to the matching specificity. The S/N threshold allows the exclusion of 2D peaks with spectral information deteriorated by the noise contribution, while the MS similarity threshold applies additional constraints to retention times alignment and improves matching specificity.

**Fig. 1:**
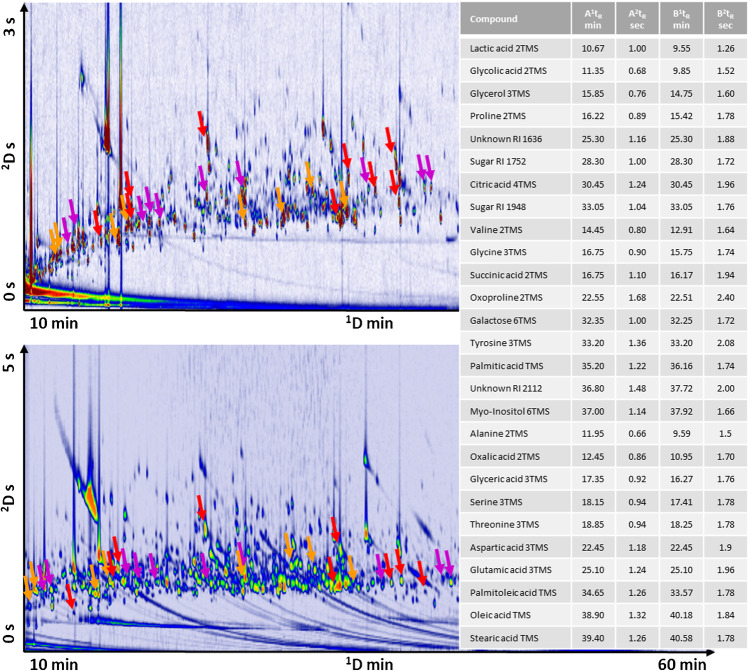
Contour plot of a reference saliva sample with the peaks used for S/N and DMF threshold highlighted according to their percent response: orange for peaks with % response relative to the IS 1,4-dibromobenzene > 0.3 (lactic acid 2TMS, glycolic acid 2TM, glycerol 3TMS, proline 2TMS, unknown with RI 1636, sugar with RI 1752, citric acid 4TMS, sugar with RI 1948), red for peaks with % response relative to the IS > 0.15 and < 0.3 (valine 2TMS, glycine 3TMS, succinic acid 2TMS, oxoproline 2TMS, galactose 6TMS, tyrosine 3TMS, palmitic acid TMS, unknown with RI 2112, myo-inositol 6TMS), and violet for peaks with % response relative to the IS < 0.15 (alanine 2TMS, oxalic acid 2TMS, glyceric acid 3TMS, serine 3TMS, threonine 3TMS, aspartic acid 3TMS, glutamic acid 3TMS, palmitoleic acid TMS, oleic acid TMS, stearic acid TMS)

To achieve the highest consistency in cross-alignment, a sub-group of analytes within those detected in the saliva samples was selected. For low-intensity and trace peaks, in particular, spectral quality fluctuations were hypothesized based on the variances in the absolute response [44]. Saliva 2D contour plots were carefully inspected and candidate 2D peaks with suitable characteristics were selected and grouped into three classes as a function of their normalized responses (i.e., TIC current/detector response normalized to that of the IS). Analytes used for performance evaluation are highlighted in an exemplary 2D contour plot illustrated in Fig. 1. The class “high” includes 2D peaks with a normalized response above 0.3, for “medium” it was between 0.15 and 0.3, and for “low” it was below 0.15.

Inconsistent MS spectral fingerprints may result in false negative matches (i.e., type-II errors when a peak that is present is not matched) for 2D peaks having S/N values lower than 50, as shown by Stilo et al. [16] since when it comes to these peaks, neither the reference nor the peak spectra are consistent enough to convey reliable information for identity confirmation. Regarding the spectral similarity threshold, a total of 20 combinations of processing parameters were tested and the number of false positive (i.e., type-I error when a peak that is not present is matched) and false negative (type-II error) matches were computed. The range of variation for S/N threshold was between 0, thus without any filter on the peak spectral signature, and 200, which was the maximum value still enabling the detection of low-intensity analytes. S/N was arbitrarily step-wise varied by 50 counts within the range. Spectral similarity, by DMF, was step-wise varied by 100 counts between the lowest value of 600 and the highest 900. The range was chosen based on previous studies [9, 16] which indicated that template matching accuracy for targeted peaks achieves its maximum with DMF around 750 if accompanied by S/N threshold values for reference template peaks above 100. In the current dataset, due to the different MS analyzers used (i.e., a TOFMS vs. qMS adopted in the Kiefl et al. [9]), the range 600–900 has been set to check the actual accuracy achievable by TOFMS detection.

Results are reported in Table 1: with DMF between 800 and 900, a significantly higher number of false negative (FN) matches occurred; type-II error mainly affects medium and low-intensity analytes, as expected. All the combinations with DMF of 800 and 900 were therefore excluded. At the same time, using a DMF of 600 increased the number of false positive (FP) matches (type-I errors), thus leading to the exclusion of such combinations of parameters. By focusing on the five combinations adopting a DMF of 700, the ones with S/N thresholds of 100, 150, and 200 were excluded because of the high rate of FN matches, of 6%, 11%, and 15%, respectively. The same results were achieved with “DMF 700 & S/N 0” and “DMF 700 & S/N 50”.

**Table 1:** Percent of false positive (FP) and false negative (FN) matches for tested combinations of S/N and DMF values used in the reliable template construction. Results are shown as FP and FN percentages for each of the three analytes classes (high, medium, and low intensity). The total corresponds to the averaged value taking in consideration the number of analytes computed in each class. “High” includes 2D peaks with a normalized response above 0.3, “medium” between 0.15 and 0.3, and “low” below 0.15

Class	%FN	%FP	%FN	%FP	%FN	%FP	%FN	%FP
	**DMF600_S/N0**		**DMF700_S/N0**		**DMF800_S/N0**		**DMF900_S/N0**	
**High**	0.00	4.17	0.69	0.00	1.39	0.69	18.06	6.25
**Medium**	1.23	1.23	4.32	0.00	14.81	0.62	72.22	0.00
**Low**	0.56	10.00	2.22	0.00	30.00	0.00	92.78	0.56
**Total**	0.62	5.35	2.47	0.00	16.46	0.41	63.79	2.06
	**DMF600_S/N50**		**DMF700_S/N50**		**DMF800_S/N50**		**DMF900_S/N50**	
**High**	0.00	2.08	0.69	0.00	1.39	0.69	18.06	4.86
**Medium**	2.47	1.85	4.32	0.00	14.81	0.62	72.22	0.00
**Low**	1.11	8.33	2.22	0.00	30.00	0.00	92.78	0.56
**Total**	1.64	4.32	2.47	0.00	16.46	0.41	63.79	1.65
	**DMF600_S/N100**		**DMF700_S/N100**		**DMF800_S/N100**		**DMF900_S/N100**	
**High**	0.00	2.08	0.69	0.00	1.39	0.69	18.06	4.86
**Medium**	4.32	1.85	7.41	0.00	15.43	0.62	72.22	0.00
**Low**	7.78	8.33	10.00	0.00	30.00	0.00	92.78	0.56
**Total**	1.90	4.32	6.38	0.00	16.67	0.41	63.79	1.65
	**DMF600_S/N150**		**DMF700_S/N150**		**DMF800_S/N150**		**DMF900_S/N150**	
**High**	0.00	2.08	1.39	0.00	1.39	0.69	18.06	4.86
**Medium**	7.41	1.85	11.11	0.00	16.05	0.62	72.22	0.00
**Low**	12.78	8.33	18.89	0.00	30.56	0.00	92.78	0.56
**Total**	2.01	4.32	11.11	0.00	17.08	0.41	63.79	1.65
	**DMF600_S/N200**		**DMF700_S/N200**		**DMF800_S/N200**		**DMF900_S/N200**	
**High**	0.00	2.08	2.08	0.00	2.08	0.69	18.06	4.86
**Medium**	10.49	1.85	16.05	0.00	19.14	0.62	72.22	0.00
**Low**	18.89	8.33	25.56	0.00	31.67	0.00	92.78	0.56
**Total**	2.18	4.32	14.81	0.00	18.72	0.41	63.79	1.65

Results comply with previous studies and related pieces of evidence on the crucial role of processing parameters in the construction of a reliable template [16, 22, 43]. However, in the current investigation, spanning a wider dynamic range of responses, the challenge posed by trace and sub-trace peaks (i.e*.*, “low” group of tested analytes) was effectively handled by lowering the S/N threshold. To note, a S/N of 0 would result in the detection of too many 2D peaks in noisy regions because of false detections. Based on the outcome of this exploration, the combination of S/N threshold of 50 and DMF of 700 was chosen to further process saliva samples’ raw data and to build a reliable template for the effective realignment of datasets.

### Dataset A—Saliva samples from diet intervention with Maillard reaction products–rich food

Optimized data processing parameters from the “Template construction parameters” section were used to generate a reliable template, thus created with peaks present in 50% + 1 of the chromatograms (*n* = 25). The list of reliable peaks (including both targeted and untargeted peak features) is reported in Table 2. The total number of reliable peaks observed was 68, 43 of which were putatively identified with the aid of *I*^*T*^ and spectrum similarity, 7 were recognized as carbohydrates but their identity was not univocally assigned, while 18 could not be associated with confidence for any putative identity.

**Table 2:** List of reliable template analytes of setup A with ^1^D and ^2^D retention time with their respective experimental *I*^*T*^. Discriminating analytes between the two diets in dataset A are listed with a variable importance in the projection (VIP) scores greater than 1. VIPs calculated by PLS-DA on normalized responses preprocessed via autoscaling

Template peak	^*1*^*t*_*R*_ min	RSD%	^*2*^*t*_*R*_ sec	RSD%	Exp *I*^*T*^	VIP
Boric acid 3TMS	9.80	0.45	0.43	2.29	714	< 1
Ethanolamine 2TMS	10.53	0.41	0.52	5.20	758	< 1
Glycolic acid 2TMS	11.35	0.24	0.67	5.15	809	< 1
Alanine 2TMS	11.93	0.36	0.67	4.37	844	< 1
Oxalic acid 2TMS	12.46	0.21	0.87	3.54	877	< 1
Unknown 1140	12.73	0.31	0.85	2.74	893	< 1
Hydracrylic acid, 2TMS derivative	12.74	0.29	0.76	2.88	894	< 1
Benzyl alcohol TMS	13.07	0.27	0.82	3.95	912	< 1
Hydroxybutyric acid 2TMS	13.11	0.28	0.73	5.38	914	< 1
Butanoic acid 2TMS (isomer)	13.27	0.27	0.74	3.44	922	1.47
Unknown 1191	13.84	0.28	0.72	4.00	951	< 1
1,4-Dibromobenzene (IS)	14.18	0.28	1.05	3.14	969	< 1
Valine 2TMS	14.45	0.24	0.80	4.58	983	< 1
Butanoic acid 2TMS	14.81	0.25	0.86	3.80	1001	1.69
Ethanolamine 3TMS	15.71	0.21	0.77	4.32	1041	< 1
Leucine 2TMS	15.84	0.92	0.86	4.07	1047	< 1
Glycerol 3TMS	15.85	0.29	0.77	4.01	1047	< 1
Proline 2TMS	16.52	0.28	0.93	3.27	1077	1.52
Succinic acid 2TMS	16.76	0.26	1.09	3.28	1088	< 1
Glycine 3TMS	16.76	0.29	0.90	3.96	1088	< 1
Glyceric acid 3TMS	17.34	0.29	0.93	3.65	1112	< 1
Serine 3TMS	18.13	0.29	0.95	3.70	1144	< 1
Butanetriol 3TMS	18.16	0.29	0.84	4.08	1145	< 1
Threonine 3TMS	18.88	0.28	0.94	3.84	1175	< 1
6-Aminocaproic acid 2TMS	18.99	0.27	1.07	2.42	1179	< 1
Butanoic acid, 3 TMS	20.02	0.29	0.98	3.11	1220	< 1
Unknown 1468	20.82	0.29	0.90	4.28	1249	< 1
Malic acid 3TMS	21.59	0.28	1.13	3.16	1278	< 1
Unknown 1520	22.26	0.65	1.04	3.76	1303	< 1
Aspartic acid 3TMS	22.46	0.30	1.18	2.67	1311	1.83
Oxoproline 2TMS	22.53	0.28	1.68	2.29	1313	1.27
Erythronic acid 4 TMS	23.29	0.29	0.99	2.78	1342	< 1
Creatinine 3TMS	23.55	0.28	1.22	3.18	1351	< 1
Unknown 1575	23.72	0.29	1.00	3.61	1357	< 1
Hydroxyglutaric acid 3TMS	23.90	0.27	1.19	3.26	1364	1.79
Glutamic acid 3TMS	25.10	0.28	1.23	2.76	1408	2.19
Unknown 1634	25.30	0.28	1.15	3.32	1415	< 1
Phenylalanine 2TMS	25.36	0.28	1.31	2.79	1417	< 1
Unknown 1636	25.37	0.28	0.94	4.29	1418	< 1
Sugar 1700	26.96	0.63	0.95	4.10	1475	< 1
Xylitol 5TMS	28.10	0.25	0.93	3.21	1516	< 1
Sugar 1752	28.31	0.39	0.98	3.82	1524	1.31
Hydrocinnamic acid	28.68	0.25	1.39	2.45	1537	< 1
Unknown 1782	29.09	0.40	1.34	2.62	1552	< 1
Unknown 1784	29.15	0.25	1.07	3.97	1555	< 1
Ribonic acid 5TMS	29.31	0.21	1.03	3.53	1561	< 1
Unknown 1808	29.69	0.26	1.00	3.02	1575	< 1
Citric acid 4TMS	30.45	0.23	1.23	3.50	1603	1.98
Sugar 1871	31.28	0.26	1.08	2.97	1634	1.27
Unknown 1881	31.49	0.28	1.21	2.87	1643	< 1
Unknown 1895	31.86	0.26	1.06	3.41	1657	< 1
Galactose TMS	32.37	0.16	0.99	3.09	1676	< 1
Sugar 1928	32.60	0.21	1.02	4.09	1685	< 1
Sugar 1948	33.05	0.28	1.04	3.65	1702	1.09
Tyrosine 3TMS	33.18	0.25	1.35	2.37	1707	< 1
Palmitoleic acid TMS	34.66	0.24	1.26	2.72	1765	< 1
Palmitic acid TMS	35.19	0.24	1.23	3.58	1786	1.01
Unknown 2112	36.77	0.24	1.47	3.10	1851	< 1
Myo-inositol 6TMS	36.97	0.25	1.12	3.25	1859	< 1
Unknown 2123	37.02	0.25	1.35	3.54	1861	< 1
Oleic acid TMS	38.88	0.23	1.31	3.07	1940	< 1
Stearic acid TMS	39.39	0.23	1.26	4.49	1961	1.04
Unknown 2361	41.91	0.26	1.84	2.47	2073	< 1
Unknown 2384	42.37	0.24	1.17	3.26	2094	< 1
Sugar 2437	43.31	0.32	1.24	3.36	2138	1.08
Sugar 2446	43.53	0.28	1.12	2.82	2148	1.77
Unknown 2654	47.29	0.26	1.17	3.30	2334	< 1
Unknown 2680	47.71	0.29	1.42	6.80	2355	< 1

**Fig. 2:**
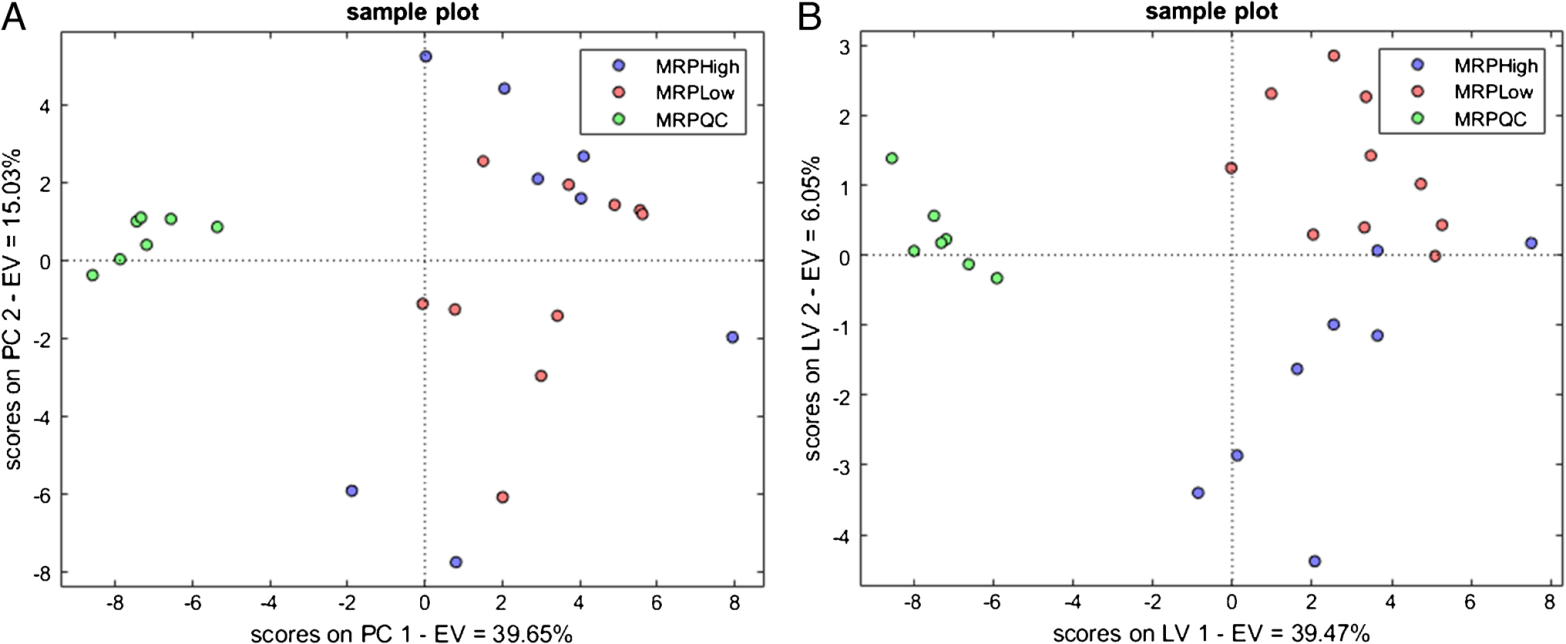
**A** PCA score plot of dataset A reliable peaks (25 samples × 68 variables) after mass spectral total useful signal (MSTUS) on included blob volume normalization and after *Z*-score normalization, displaying QC samples (green), high MRPs (blue), and low MRPs (red) diets. **B** PLS-DA score plot of dataset A reliable peaks (25 samples × 68 variables) displaying QC samples (green), high MRPs (blue), and low MRPs (red) diets

Unsupervised and supervised statistics were conducted on both reliable peaks and peak regions after normalization and scaling of the absolute response to find distinctive patterns of metabolites associated with diet intervention with MRPs-rich food. The PCA on reliable peaks (25 samples × 68 variables)—score plot shown in Fig. 2A—explained the differences between the QC samples and the saliva samples from MRPs-rich/low diets. Even though no natural clusterization was evident between the two conditions under study, a PLS-DA was conducted to maximize the differences for descriptive modeling as well as for discriminative variable selection—score plot shown in Fig. 2B. The latter objective was achieved by looking at the variable importance in the projection (VIP) scores, and by considering discriminating variables as those with a VIPs > 1. Glutamic acid, citric acid, aspartic acid, hydroxyglutaric acid, butanoic acid, oxoproline, stearic acid, and palmitic acid were identified as the most discriminant, together with five carbohydrates and 7 unknowns (i.e., also referred to as knowns unknowns according to Stilo et al. [45])—Table 2. The same approach was conducted with peak regions with comparable results.

Both stearic and palmitic acids were previously identified as possible markers of dietary lipid intake and correlated with obese patients affected by hepatic steatosis [46, 47] and showed a relative increase in diabetic patients and periodontal diseases [48]. However, the intake of the high-MRP diet was accompanied by a higher intake of fats. Butyric acid, a locally produced metabolite of pathogenic periodontal bacteria, has been shown to cause caspase-dependent apoptosis in gingival fibroblasts and could potentially operate as a chemotaxonomic indicator of bacterial metabolism in the oral environment [49]. Five non-identified monosaccharides were typical of high-MRP diets, as expected. The non-identified monosaccharides leave space for speculations. Beside MRPs, the high-MRP diet contained more sugar and starch as expected from the food questionnaires. Structural similar sugar metabolites such as 3-deoxyglucosone (3-DG) or other dicarbonyl compounds may be taken into account. The presence of carbonyls, in particular the reactive dicarbonyl species, e.g., methylglyoxal, reacts with amino compounds of endogenous tissues in the body, leading to that what is known as “carbonyl stress,” which causes an increase in protein and DNA alteration that contribute to cell and tissue malfunction in aging and illness [50, 51]. The role of dietary MRPs in this scenario is not yet clear.

### Template transformation for reliable template matching between severely misaligned patterns

Experimental settings between dataset A and dataset B create large pattern differences with inconsistent misalignments due to the concurrent effect of several parameters: (a) *P*_M_ 3 s vs. 5 s, (b) different column lengths/dimensions, (c) carrier gas nominal flow differences (1.3 mL/min vs. 1.6 mL/min) and variable average velocities (19.0 cm/s and 201.9 cm/s vs. 16.3 cm/s and 164.9 cm/s for ^1^D and ^2^D, respectively), (d) oven temperature programming, and (e) MS detector optimization. Figure 1A, B shows the contour plot of saliva metabolites from dataset A (Fig. 1A) and dataset B (Fig. 1B). Highlighted compounds are those considered for the template parameters optimization (the “Template construction parameters” section).

To guide the correct strategy for template transform, e.g., global or local, affine or low-degree polynomial function, pattern discrepancies generated by the two experimental setups were computed. To simplify the process, the reference standard mix including selected amino acids, organic acids, carbohydrates, and additional salivary metabolites, analyzed within datasets A and B, was cross-aligned in the temporal domain.

**Fig. 3:**
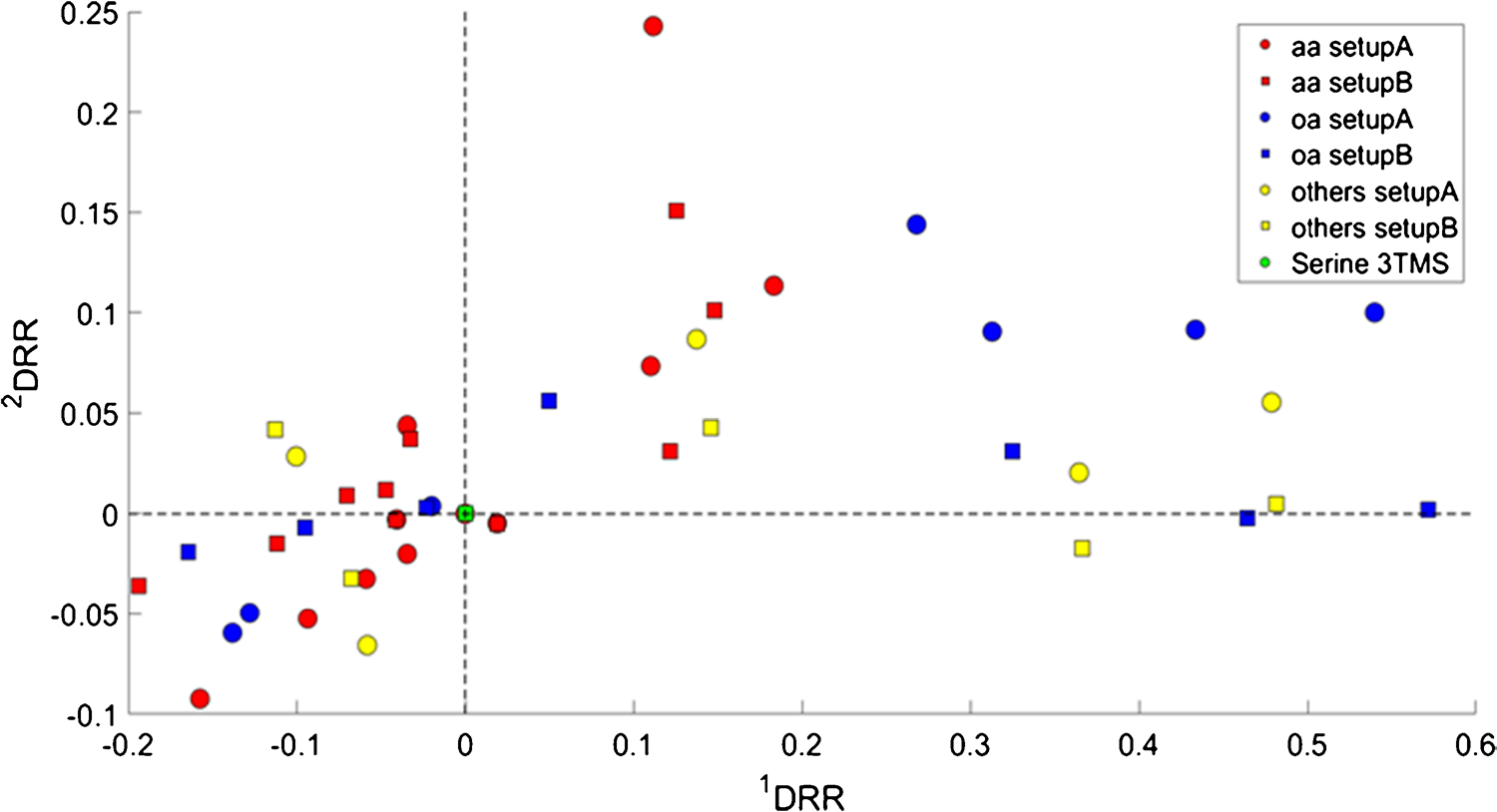
Scatter plot resulting from the relative retention on both 1^st^ and 2^nd^ dimensions (^1^DRR and.^2^DRR, respectively) of targeted peak analytes from the two setups relative to serine 3TMS (green): amino-acids (red), organic acids (blue), other metabolites (yellow)

Figure 3 shows the pronounced, non-linear ^1^D and ^2^D retention time shifts generated by the experimental conditions. In particular, analytes are represented in the Cartesian space corresponding to the ^1^D and ^2^D retention, with relative position over two reference peaks. Serine 3TMS derivative, which elutes in the middle retention times region of the chromatographic plane, was assigned as a centroid, while stearic acid, the last-eluting analyte, was used to normalize the ^1^D relative position [38, 52]. ^1^D and ^2^D relative retention (RR) values were calculated by Eqs. 1 and 2:

1 $${}^{1}DRR={(}^{1}{{t}_{R{1}^{-}}}^{1}{t}_{R Serine, 3TMS}){/}^{1}{t}_{R Stearic acid, TMS}$$

2 $${}^{2}DRR={(}^{2}{{t}_{R{i}^{-}}}^{2}{t}_{R Serine, 3TMS}){/}^{1}{P}_{M}$$

where ${}^{\mathit1}t_{\mathit R\mathit i}$  corresponds to the ^1^D retention time expressed in minutes for target peak *i*, ${}^{\mathit2}{\mathit t}_{\mathit R\mathit i}$ corresponds to the ^2^D retention time expressed in seconds for the target peak *i*, and *P*_M_ is the modulation period. The two patterns showed dramatic misalignments mainly due to changes (*a*), (*b*), (*c*), (*d*), which led to opposite elution order in both ^1^D and ^2^D across the first and third quadrant for compounds belonging to the same chemical class for both amino acids and organic acids respectively indicated in red and blue.

To define the best strategy to solve the misalignment issue, QC samples from the two datasets were compared. Rules for template peak thresholds and reference spectra were then applied to build a reference targeted template for known analytes. By examining patterns from dataset A, a template of 52 2D peaks was created; reference peaks inclusion was restricted to analytes with an S/N of 50 or higher; reference spectra were taken from peak spectrum; the MS constraint was set at 700 DMF and 700 reverse match factor (RMF).

Different template transformation strategies, within those available in the software, were tested to solve the significant misalignment between patterns on datasets A and B. They are (a) *local match-and-transform* algorithm which applies the affine transform function to locally realign template peaks to candidate peaks in the chromatogram; (b) *translation-and-scale algorithm* which applies the affine transform-and-rescale according to positively matched peaks; (c) *global polynomial transform* which applies 2^nd^ order degree polynomial functions for template transformation; and (d) supervised *multi-centroid transformation* which includes the analyst supervision guiding template subdivision and local translation followed by a local affine transformation.

To obtain a genuine representation of the real-world application, instead of evaluating the potential reliable template transformations over the standard mix of amino acids, organic acids, carbohydrates, and selected salivary metabolites, QC samples from dataset B, consisting of saliva collected from healthy Italian volunteers, were used. This allows assessment of the quality of the transformation in a chromatographic plane where the complexity of a real-world sample consists of a greater number of 2D peaks and interferents, a condition that would not be considered if a standard mixture was used.

The targeted template obtained from dataset A with the parameters evaluated in the “Template construction parameters” section was applied over four QC analyses from dataset B (i.e., REF1, REF2, REF3, and REF4). A graphical schematization of the workflow is illustrated in Figure SF1.

Strategy 1 involved the following procedure: the template was linearly transformed on the ^2^D to realign the IS peak (1,4-dibromobenzene). Iterating the process of matching the template and transform it based on the matched locations allows the template to be tailored to the new pattern. This procedure gradually increases the number of available alignment points, enhancing the quality of the global template transformation at every stage up to a plateau. For instance, with sample REF1 the first matching ratio resulted in 46.15% of positive matches corresponding to 24/52 matched peaks. After reiterating the transform and match for 3 additional times, the total number of matched peaks resulted 28/52 corresponding to 53.84% positive matches. Strategy 2 is similar to 1, but includes exponential scaling transformations, while strategy 3 includes polynomial ones. The latter was effective in solving complex realignment issues like with translated methods from thermal to flow modulated platforms and showed positive results when realigning chromatograms acquired with different operative pressures [16, 22].

Strategy 4 involves the following steps: (a) split the reference template into multiple subsections, (b) identify a reliable peak with high intensity for each subsection as a centroid, (c) manually realign the centroids, and (d) reiterate the match-and-transform procedure until a plateau. To realign the two datasets, 1,4-dibromobenzene, proline 2TMS, oxoproline 2TMS, and palmitic acid were chosen as local realignment points. Template matching results for the four approaches are summarized in Table 3. Results with a template consisting of only reliable peaks showed that the manual multi-centroid transformation (strategy 4) performed equally to the global polynomial transformation (strategy 3), both superior to the local *match-and-transform* (strategy 1) and the *affine* algorithms (strategy 2). The main shortcoming of reiterated global polynomial transformation is the possible deformation of the peak regions template on the chromatographic plane as a result of the overfitting, thus making it the optimal solution with templates constituted by only reliable peaks.

**Table 3:** Template matching results for test template from dataset A applied to test dataset 2 samples after applying template transformation algorithms

	Match-and-transform		Translation and scale		Polynomial 2^nd^ order		Multiple centroid match-and-transform	
	Peaks no	%	Peaks no	%	Peaks no	%	Peaks no	%
**Test 1**	28	53.85	33	63.46	33	63.46	33	63.46
**Test 2**	29	55.77	32	61.54	34	65.38	34	65.38
**Test 3**	26	50.00	29	55.77	31	59.62	31	59.62
**Test 4**	26	50.00	32	61.54	33	63.46	33	63.46
**Mean**	27.25	52.40	31.50	60.58	32.75	62.98	32.75	62.98
**SD**	1.50		1.73		1.26		1.26	
**RSD%**	5.50		5.50		3.84		3.84	

**Fig. 4:**
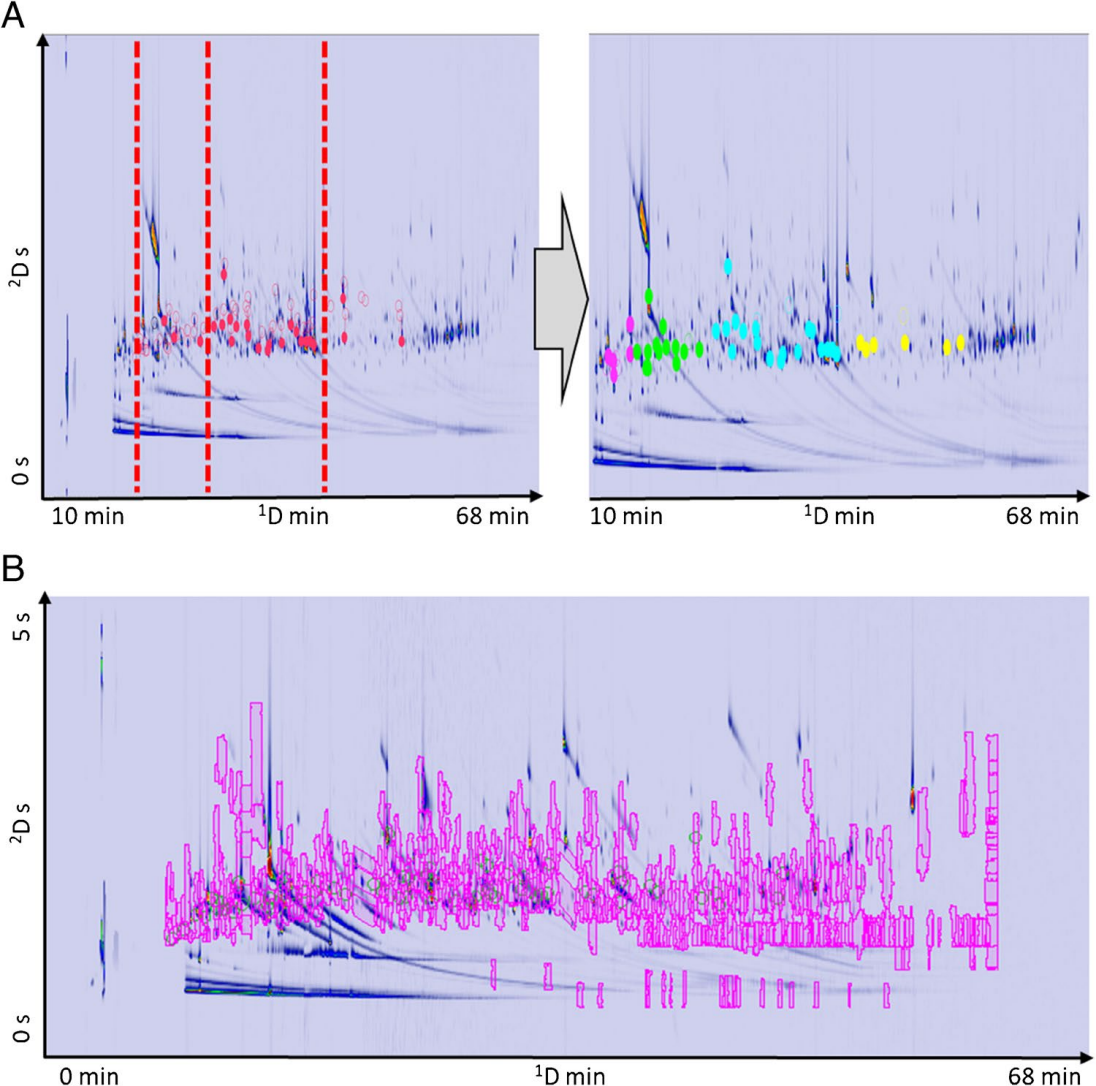
**A** Testing template resulting from standard mixture transformation with manual multi-centroid approach (strategy 4). **B** Feature template from setup A transformed with manual multi-centroid approach on a reference sample from setup B

To cross-aligning datasets A and B, strategy 4 was successfully adopted with the following template transformation settings: 0–14.5 min was shifted of − 18 datapoints (dp) on ^1^D and 0 dp on ^2^D, 14.5–21.5 min was shifted of − 13 dp on ^1^D and 0 dp on ^2^D, 21.5–34 min was shifted of 0 dp on ^1^D and − 6 dp on ^2^D, and 34–66 min was shifted of + 11 dp on ^1^D and − 16 dp on ^2^D. Afterward, strategy 2 was adopted to finely adapt the subregions; the transformation described as strategy 4 is shown on the testing template in Fig. 4A, and the final template applied over a test sample is illustrated in Fig. 4B.

### Response normalization for consistent cross-comparison between datasets

The reduction of features into a similar range, known as data normalization, is a crucial pre-processing step that prevents larger numeric feature responses from dominating smaller numeric ones. The major objective is to reduce the bias of those features in pattern classes that contribute numerically more than others, so that the variables are given equal weight when computing statistical analysis but still avoiding the implication that these features are equally relevant [53, 54].

Many strategies are available to model the distribution of the original values into a new set of values. Within these, the most common mathematical ones consist of min–max normalization, *Z*-score normalization, and logarithmic transformation. The min–max normalization rescales the data within a new minimum and maximum value preventing highly concentrated peaks from dominating the other ones in a dataset with a significant min–max difference [55]. With *Z-*score normalization, each variable’s mean is subtracted from the data values individually and followed by dividing data values by standard deviation to give the variables a variance of one [56]; compared to min–max normalization, *Z-*score normalization is less prone to create bias deriving from outliers. The latter, i.e., logarithmic transformation, consists of replacing each value in the dataset with its logarithm, thus allowing to make skewed distributions more symmetric [57].

Besides mathematical approaches, such as those listed above, scaling transformation strategies can be also applied to the raw response data. In the mass spectral total useful signal (MSTUS) [58], for example, each variable is normalized over a selection of ions/features (e.g., total volume of reliable peaks and peak regions features) to get a more accurate normalization by avoiding the inclusion of noise or artifact peaks [59, 60].

Moreover, before data mining, missing values can be replaced with random values of at least half of the smallest response since they are typically caused by the signal intensity being below the LOD; the replacement with random values instead of 0 or a fixed number is generally performed to avoid bias-introduction for further unsupervised and supervised statistical approaches.

Absolute response and background noise intensity are directly impacted by MS detector response oscillations caused by MS tuning, optimization, or other causes. During this study, datasets A and B were acquired in a 2-year time frame; thus, differences in terms of MS performances are expected. Output signals exhibited different absolute TIC responses (i.e., different actual sensitivity) and background noise intensities. MS performances between the two datasets were evaluated by comparing two QC analyses picked from those collected during the two analytical batches. Background noise was sampled in the middle of the chromatogram and reported an average intensity of 8484 counts (^1^D RSD% = 2.91%, ^2^D RSD% = 1.77%) for the dataset A and 24,522 counts (^1^D RSD% = 22.16%, ^2^D RSD% = 8.86%) for dataset B before any background correction was applied. After the baseline removal, the noise intensity averaged 127 and 2000 counts, respectively. Experimental results indicate that dataset B had a greater absolute noise (2.9 times) compared to dataset A while background noise correction greatly affected dataset A reducing the noise signal by 66.5 times compared to the 12.26 times of the dataset B. The number of detected peaks in dataset A with S/N > 50 was 137, compared to 498 in dataset B, indicating a higher relative sensitivity of the method in the second case.

**Fig. 5:**
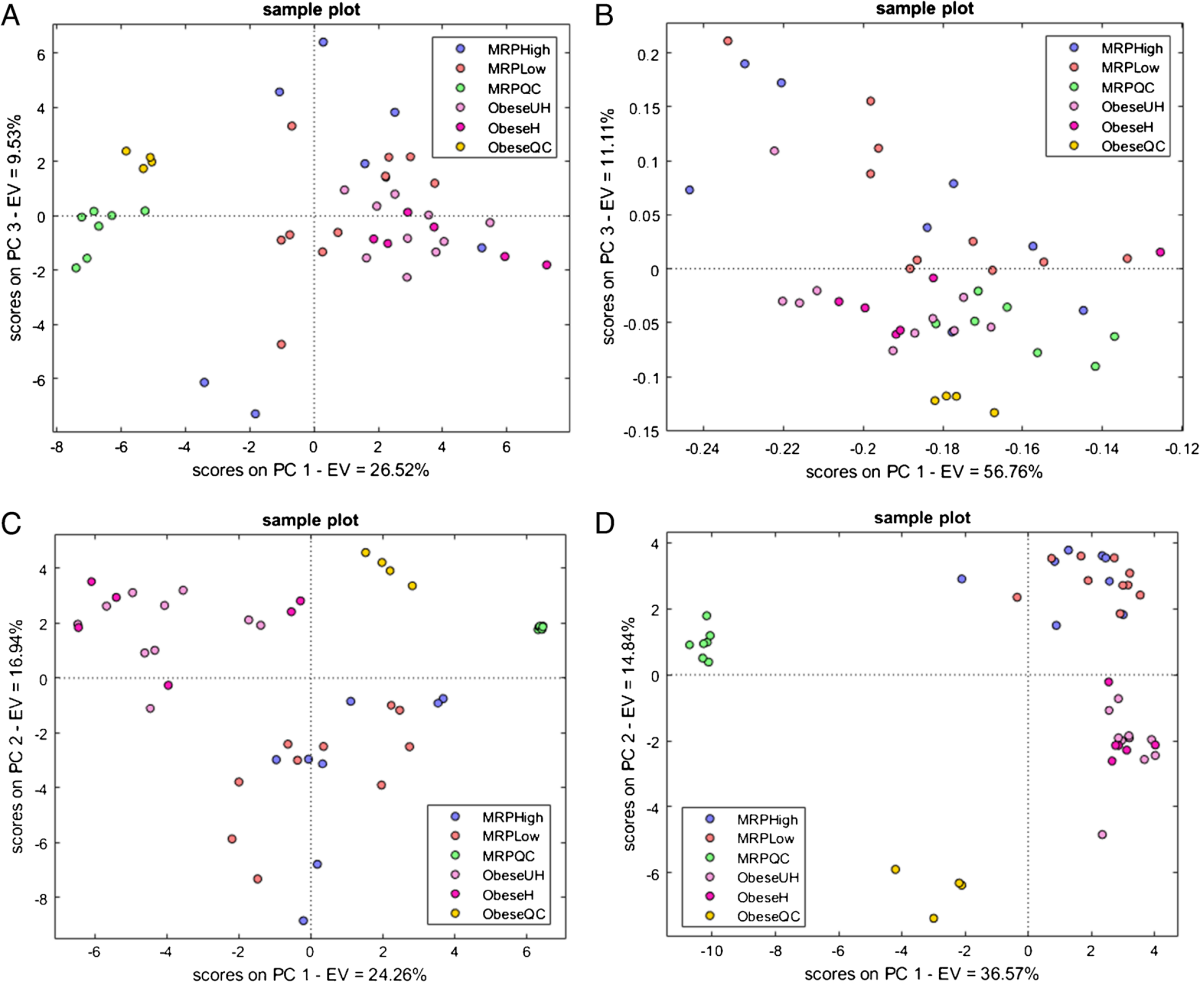
**A** PCA score plot of the merged datasets A and B after realignment and normalization on mass spectral total useful signal (MSTUS) on included blob volume. Score plot resulting from reliable peaks (45 samples × 64 variables) after *Z*-score normalization displaying dataset A QC samples (green), high MRPs (blue), low MRPs (red) diets, dataset B QC samples (yellow), healthy obese (purple), and unhealthy obese (pink). **B** PCA score plot after realignment and MSTUS normalization without *Z*-score normalization. **C** PCA score plot without MSTUS normalization and with *Z*-score normalization. **D** PCA score plot with log scale and z-score normalization

Different combinations of data pretreatment were performed on the fused dataset (A + B), after which peaks and peak region features were mined with unsupervised statistics (PCA). Results are illustrated in Fig. 5. Figure 5A shows the combination of MSTUS normalized responses on the included 2D peaks volume with *Z-*score normalization on the resulting matrix. Although no differences arise between the healthy and unhealthy obese patients from dataset B and the diets high and low in MRPs foods from dataset A, the two datasets are considered comparable given the realignment of the QC samples obtained from the two datasets. On the other hand, Fig. 5B shows the issue arising by omitting the *Z-*score normalization after MSTUS normalization: no clear clustering is achieved due to an imbalanced min–max distribution of the data matrix. Figure 5C and D both show the necessity in metabolomics studies to apply MSTUS normalization when comparing different datasets: despite having different approaches (raw responses and logarithmically scaled responses respectively), comparison between the samples cannot be performed as the detector performance fluctuations heavily affect the quality of the data. It can be concluded that such issues/inconsistencies can be only overcome with MSTUS normalization.

**Fig. 6:**
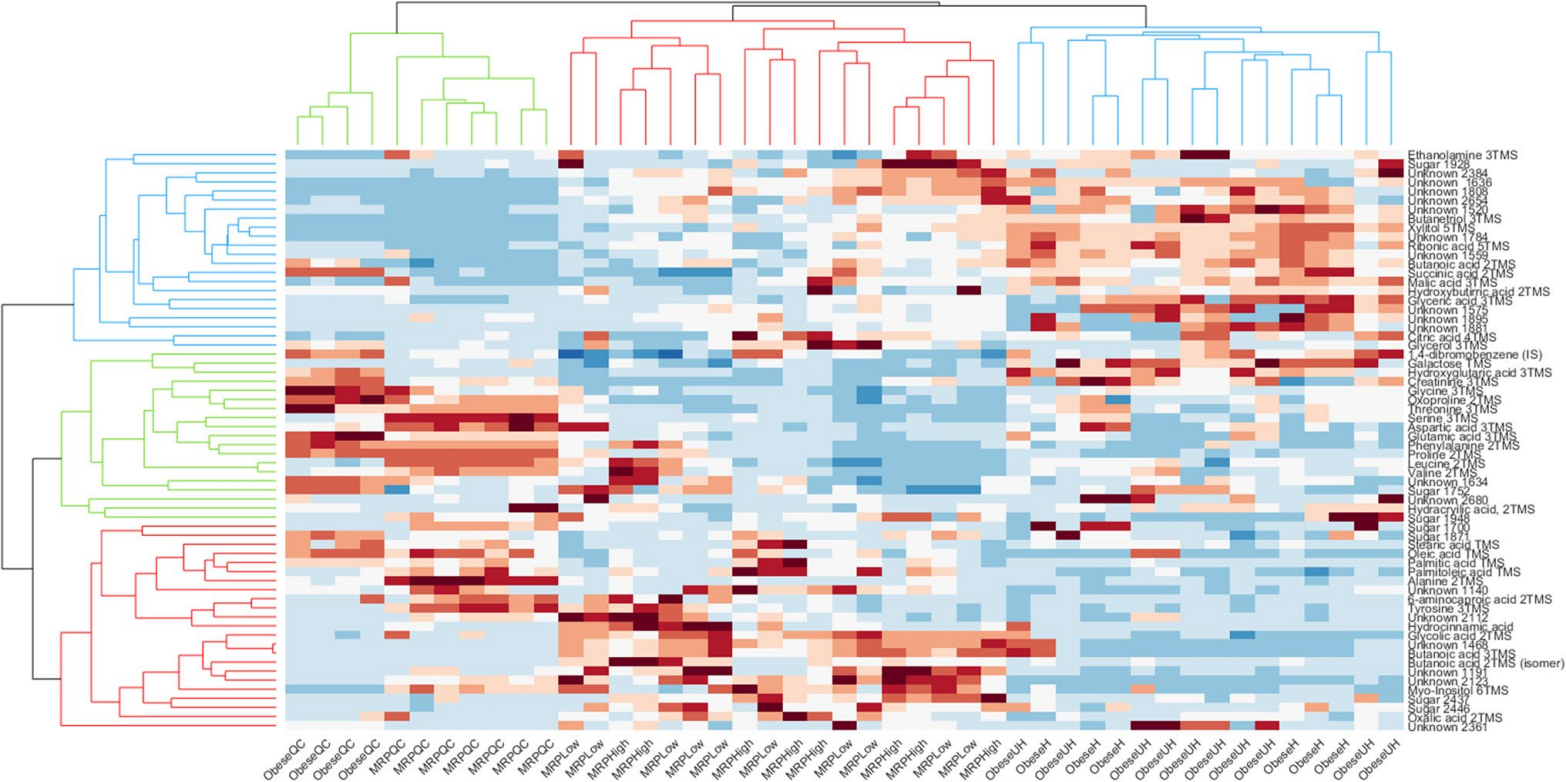
Heatmap of the merged datasets A and B after realignment and normalization on percent responses; dendrogram built with Spearman’s correlation for both rows and columns. QC from datasets A and B are clustered together, high and low MRPs samples are clustered together, as well as healthy and unhealthy obese patients. MRPs and obese samples resulted more similar compared to QC samples

The hierarchical clustering (HC) of the fused dataset obtained on MSTUS normalized responses on the included blob volume is illustrated in Fig. 6 (data available on request—see “Data availability”). Interesting biological outcomes can be retrieved from this comparison: saliva samples from QC samples were richer (higher relative distribution) in amino acids such as oxoproline, aspartic acid, and glutamic acid, which were found to be discriminant variables characteristic of the low MRP diet group, as a witness of the different eating habits of the two populations. On the other end, both metabolically healthy and unhealthy obese patients were clustered together and characterized by higher levels of butyric acid and hydroxybutyric acid, which were previously confirmed as obesity markers detected in both urine and blood [61]. Further investigations on dataset 2 were already carried out by Cialiè Rosso and coworkers [14] and will not be further discussed here.

## Conclusions

This paper examined 2D data processing strategies based on pattern recognition algorithms, which are capable of overcoming 2D pattern severe misalignments and detector response fluctuations generated in analyses acquired within wide time frames. The strategy described in the current paper allows the creation of a template capable to adapt to different datasets without the necessity to reiterate the preprocessing of the additional batches and to preserve the information encrypted in the metadata (i.e., reliable peaks) to extract biological information once the data fusion is performed. Focusing on the chromatographic aspect, severe misalignments derived by the concurring effect of different analytical setups (different nominal flows and carrier velocities, variations in the actual pressure drop, column dimensions, and *P*_M_) can be solved by a relatively simple strategy consisting of a manual multi-centroid transformation. Regarding the response fluctuation caused by differences in the detector performances, the most promising strategy to cross compare different datasets was MSTUS normalization based on the included blob volume followed by a *Z-*score normalization on the resulting matrix.

## Supplementary information

Below is the link to the electronic supplementary material.

Supplementary file1 (DOCX 32.5 KB)

## Data Availability

Data has been uploaded to the Open Science Framework (OSF) website in a dedicated repository: https://osf.io/jbdxe/?view_only=603945dcc6e547b981b3615bc1c3cf6e. The access is made available on request.
