# Correction to: Extending the breadth of saliva metabolome fingerprinting by smart template strategies and effective pattern realignment on comprehensive two‑dimensional gas chromatographic data

**DOI:** 10.1007/s00216-023-04572-3

**Published:** 2023-02-18

**Authors:** Simone Squara, Friederike Manig, Thomas Henle, Michael Hellwig, Andrea Caratti, Carlo Bicchi, Stephen E. Reichenbach, Qingping Tao, Massimo Collino, Chiara Cordero

**Affiliations:** 1 Dipartimento Di Scienza E Tecnologia del Farmaco, Universita Degli Studi Di Torino, Turin, Italy; 2 Food Chemistry, Technische Universitat Dresden, Dresden, Germany; 3 Special Food Chemistry, Technische Universitat Dresden, Dresden, Germany; 4 Computer Science and Engineering Department, University of Nebraska, Lincoln, NE USA; 5 GC Image LLC, Lincoln, NE USA; 6 Dipartimento Di Neuroscienze “Rita Levi Montalcini”, University of Turin, Turin, Italy

**Correction to:Analytical and Bioanalytical Chemistry**

**https://doi.org/10.1007/s00216-023-04516-x**

The original article contained a mistake.

Author Qingping Tao should be affiliated to affiliation 5 and author Massimo Collino should be affiliated to affiliation 6.

Affiliation 6 should be corrected to: Dipartimento Di Neuroscienze “Rita Levi Montalcini”, University of Turin, Turin, Italy

The original article and supplementary file has been corrected.

