# Supplementary material for: Extending the breadth of saliva metabolome fingerprinting by smart template strategies and effective pattern realignment on comprehensive two-dimensional gas chromatographic data
Source: Anal Bioanal Chem. 2023 Jan 12;415(13):2493–509. doi: 10.1007/s00216-023-04516-x (PMC10149478; doi:10.1007/s00216-023-04516-x)
Supplement: Supplementary file 1 — Supplementary file1 (DOCX 32.5 KB) [file 216_2023_4516_MOESM1_ESM.docx]

**Supplementary Electronic Material**

**Extending the Breadth of Saliva Metabolome Fingerprinting by Smart Template Strategies and Effective Pattern Realignment on Comprehensive Two-Dimensional Gas Chromatographic Data**

Simone Squara^1$^, Friederike Manig^2$^, Thomas Henle^2^, Michael Hellwig^3^, Andrea Caratti^1^, Carlo Bicchi^1^, Stephen E. Reichenbach^4,5^, Qingping Tao^5^, Massimo Collino^6*^, Chiara Cordero^1*^

^$^Simone Squara^1^ and Friederike Manig^2^ equally contributed to this work.

Authors’ affiliation:

^1^Dipartimento di Scienza e Tecnologia del Farmaco, Università degli Studi di Torino, Turin, Italy

^2^Chair of Food Chemistry, Technische Universität Dresden, Dresden, Germany

^3^Chair of Special Food Chemistry, Technische Universität Dresden, Dresden, Germany

^4^Computer Science and Engineering Department, University of Nebraska, Lincoln, NE, USA

^5^GC Image LLC, Lincoln, NE, USA

^6^Dipartimento di Neuroscienze, Università degli Studi di Torino, Turin, Italy

*Address for correspondence:

Prof. Chiara Cordero - Dipartimento di Scienza e Tecnologia del Farmaco, Università degli Studi di Torino, Via Pietro Giuria 9, I-10125 Torino, Italy – e-mail: chiara.cordero@unito.it; phone: +39 011 6702197

Prof. Massimo Collino - Dipartimento di Neuroscienze, Università degli Studi di Torino, Corso Raffaello 30, I-10125 Torino, Italy – e-mail: massimo.collino@unito.it; phone: +39 011 6706861

**Supplementary Figure 1 - SF1:** graphical workflow of the necessary steps to be performed to create a reliable template, the strategies to be adopted for template transformation with a misaligned dataset, and the chemometrics operations required to extract biological information from the two realigned datasets.

Step 1

**Realiable template construction (GC Image^TM^ & Image Investigator^TM^)**

- Data preprocessing (Data import, rasterization, Baseline correction, 2D peaks detection and integration)

- Set processing parameters that were evaluated in Section 3.1

- Build a reliable template of known and unknown peaks and peak regions features

Step 2

**Realiable template transformation for cross comparison with a misaligned dataset (GC Image^TM^)**

- Four candidate strategies appropriate with misalignments caused by different issues, listed from one to four in ascendent order of severity

(1) Batch effect

(2) Oven programme

(3) Pressure drops

(4) Combination variables

Local match-and-transform

algorithm

Affine transformation

algorithm

Low-degree polynomial transformation

Manual
multi-centroid transformation

- The outcome of the four transformation strategies applied over a case study where severe misalignments were observed due to the concurrent effect of multiple variables (pressure drop, column dimensions, *P_M_*) is the following: (1) achieved the realignment of 52.4% of peaks, (2) achieved 60.58%, (3) achieved 62.98% but with the distortion of the peak regions features, (4) achieved 62.98% without template distortions.

Step 3

**Data mining**

- Data pretreatment by normalization and transformation

- Handling of missing values

- Data Fusion

- Unsupervised statistics for natural clustering detection

- Supervised classification for variable selection and discriminating analytes identification

- Classification models for future samples clusterization
